# Hepatic artery infusion therapy is effective for chemotherapy-resistant liver metastatic colorectal cancer

**DOI:** 10.1186/s12957-015-0704-5

**Published:** 2015-10-09

**Authors:** Takanori Goi, Takayuki Naruse, Youhei Kimura, Daisuke Fujimoto, Mitsuhiro Morikawa, Kenji Koneri, Akio Yamaguchi

**Affiliations:** First Department of Surgery, University of Fukui, 23-3, Eiheiji-cho, Yoshida-gun, Fukui 9101193 Japan

**Keywords:** Chemotherapy-resistant liver metastasis, Colorectal cancer, Hepatic artery infusion chemotherapy

## Abstract

**Background:**

Systemic FOLFOX (folinic acid (leucovorin (LV)), 5-fluorouracil (5-FU), and oxaliplatin), FOLFIRI (LV, 5-FU, and irinotecan), or FOLFOXIRI (5-FU, leucovorin, oxaliplatin, and irinotecan) chemotherapy regimens and additional molecular-target treatments, including anti-vascular endothelial growth factor, anti-epidermal growth factor receptor, and anti-multi-kinase antibodies, have been recommended for unresectable recurrent colorectal cancers. However, no effective treatments are currently available for cases refractory to these therapies. Therefore, the development of alternative therapies is desired. In the present study, we administered and observed the effectiveness of hepatic artery infusion therapy (HAIC) in patients with unresectable liver metastatic colorectal cancers refractory to systemic chemotherapy. In addition, we observed that in an experimental system with anticancer drug-resistant colorectal cancer lines, apoptosis and cell death could be induced by increasing anticancer drug concentrations.

**Methods:**

The subjects had liver metastatic colorectal cancers that were unresponsive to systemic chemotherapy (FOLFOX/FOLFIRI) or to additional molecular-target therapies for progressive disease. Hepatic infusion tube placement was conducted according to the Seldinger method to insert a catheter with a side hole via the right femoral artery. A coiling procedure was performed to prevent drug influx into the gastroduodenal artery. Ten subjects were selected, and the results were evaluated after HAIC (5-FU and LV administered once weekly). Moreover, anticancer drug-resistant colorectal cancer lines were subsequently prepared to investigate whether increased anticancer drug concentrations could induce apoptosis or cell death.

**Results:**

Of the 10 subjects, 3 (30 %) showed partial response and 4 (40 %) showed no change according to computed tomography imaging findings obtained after hepatic artery infusion. The disease control rate was 70 %. Eight subjects had improved quality of life. Survival time ranged from 2 to 16 months (median, 9 months). Meanwhile, we found that higher anticancer drug concentrations induced apoptosis and cell death in an anticancer drug-resistant colorectal cancer cell line.

**Conclusions:**

HAIC was effective in some systemic chemotherapy-resistant colorectal cancers with liver metastases and should be considered as an effective palliative therapy. This supports the finding that apoptosis and cell death could be induced in anticancer drug-resistant colorectal cancer cells in a drug concentration-dependent manner.

## Background

Colorectal cancer is highly prevalent in Japan and Western countries, and the number of deaths resulting from malignant tumors is substantial [1–3]. Given the availability of various anticancer and molecular targeting agents during the past one to two decades, the survival prognosis of unresectable or recurrent colorectal cancer has been improved to approximately 30 months [4]. The current National Comprehensive Cancer Network (NCCN) guideline recommends a systemic chemotherapy regimen comprising a FOLFOX/FOLFIRI/FOLFOXIRI (folinic acid (leucovorin (LV)), 5-fluorouracil (5-FU), and oxaliplatin/irinotecan) regimen plus molecular-target treatments (an anti-vascular endothelial growth factor, anti-epidermal growth factor receptor, aflibercept, or anti-multi-kinase antibody therapy) for unresectable/recurrent colorectal cancers [5]. However, no effective therapies have been established for cases refractory to the recommended treatment regimens. The liver is the most frequent site of colorectal cancer recurrence [6, 7]. Such tumors will eventually lead to hepatic dysfunction and failure, frequently causing death. Accordingly, survival might be prolonged by using an effective treatment against hepatic metastatic lesions. Although transcatheter arterial chemoembolization, radiofrequency ablation, external beam radiation therapy, and hepatic artery infusion are currently considered the primarily local liver treatment modalities [8–13], hepatic artery infusion did not yield any survival benefit in a comparative study of systemic chemotherapy and therefore has not often been used [14, 15]. However, in cases of solitary hepatic tumors, hepatic artery infusion can deliver high concentrations of anticancer drugs to the tumor to yield substantial tumor shrinkage. Therefore, this modality is considered significantly useful.

In the following paragraphs, we present our findings regarding the efficacy of hepatic artery infusion for unresectable colorectal cancer metastatic liver lesions and impending hepatic failure in patients who were unresponsive to systemic chemotherapy. Furthermore, we prepared and exposed anticancer drug-resistant colorectal cancer cells to high anticancer drug concentrations and observed the induction of apoptosis and cell death.

## Methods

### Patients

The 10 subjects had progressive disease (PD), unresectable colorectal cancer that was treated with FOLFOX, FOLFIRI, anti-VEGF antibody, or anti-EGFR antibody therapy according to the guideline and hepatic metastases that were considered a prognosis-determining factor at the First Department of Surgery of the University of Fukui between 2011 and 2014 (Table 1).

**Table 1:** Patient characteristics

Age	Primary	Extra	K-Ras mut	UGT1A1	Previous chemotherapy hepatic lesions
1. 60	S	Lung	−	Wild	5FU, L-OHP, CPT-11 anti-VEGF/EGFR Ab
2. 71	D		−	Homo	5FU, L-OHP, anti-VEGF/EGFR Ab
3. 69	D		+	Wild	5FU, L-OHP, CPT-11, Anti-VEGF Ab
4. 63	T	Peritoneum	−	Wild	5FU, L-OHP, CPT-11, anti-VEGF/EGFR Ab
5. 55	R		+	Wild	5FU, L-OHP, CPT-11, anti-VEGF Ab
6. 81	C	Bone	+	Hetero	5FU, L-OHP
7. 55	R	Lung	−	Hetero	5FU, L-OHP, CPT-11, anti-VEGF Ab
8. 56	D	Lung	−	Hetero	5FU, L-OHP, CPT-11, anti-VEGF/EGFR Ab
9. 56	R		−	Wild	5FU, L-OHP, CPT-11, anti-VEGF Ab
10. 57	R		−	Wild	5FU, L-OHP, CPT-11, anti-VEGF/EGFR Ab

*C* cecum, *T* transverse, *D* descending, *S* sigmoid, *R* rectum)

**Table 2:** Results of HAIC treatment

	HAIC (times)	Response	New extrahepatic lesions	Overall survival (months)
1.	12	SD	–	5
2.	18	PR	–	9
3.	34	PR	Peritoneum, brain	12
4.	44	PR	–	16
5.	7^a^	SD	Peritoneum, lung, bone	4
6.	3^a^	PD	Lung	2
7.	16	SD	–	11^b^
8.	8	SD	–	3
9.	6	PD	Bone	9^b^
10.	6	PD	–	3^b^

^a^Discontinuation of HAIC, 5. Interstitial pneumonia, 6. Malaise

^b^Alive

**Table 3:** Tumor response

No. of patients	Response				Disease control rate (%)
	C.R.	P.R.	S.D.	P.D.	
10	0	3	4	3	70 %

**Table 4:** Patients’ complaints

Patient	HAIC	
	Pre-	Post-
1.	Difficulties with walking, leg edema, malaise	Recovered during 4 months
2.	Malaise, appetite loss	Recovered during 6 months
3.	Leg edema	Recovered during 9 months
4.	Leg edema	Recovered during 14 months
5.	Leg edema	Recovered during 2 months
6.	General fatigue, leg edema, low back pain	No change
7.	Epigastric pain, leg edema	Recovered during 6 months
8.	Leg edema	Recovered during 2 months
9.	–	–
10.	Malaise	Recovered during 3 months

**Table 5:** Toxicity (CTCAE v4.0)

	Grades 1–2	Grade 3–4
Diarrhea	0 (0 %)	0 (0 %)
Anorexia	1 (10 %)	0 (0 %)
Malaise	0 (0 %)	1 (10 %)
Neutropenia	1 (10 %)	0 (0 %)
Thrombopenia	0 (0 %)	0 (0 %)
Interstitial pneumonia	0 (0 %)	1 (10 %)

### Hepatic artery infusion therapy

**Fig. 1:**
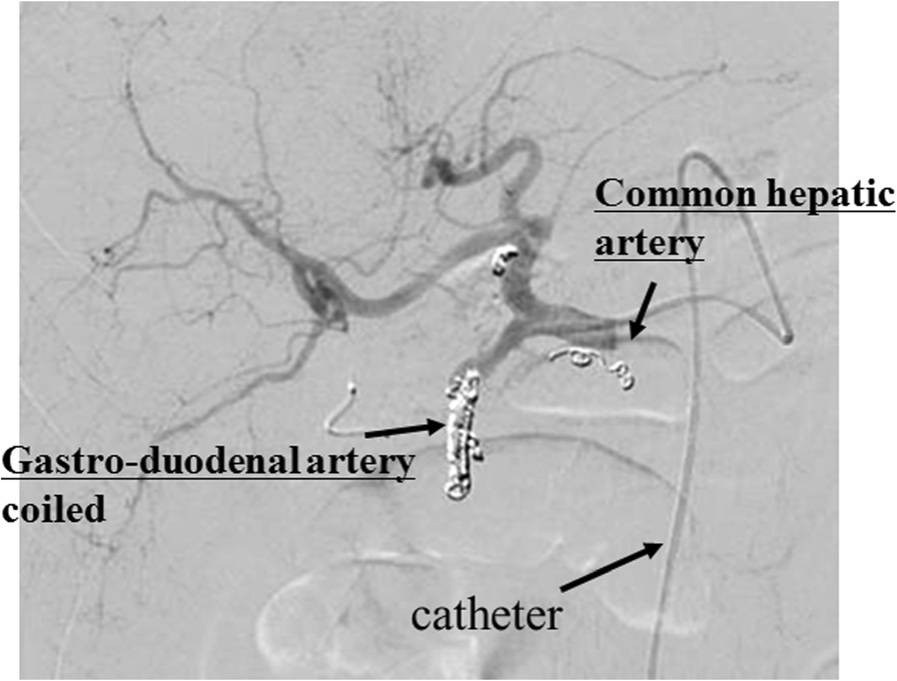
Hepatic artery infusion therapy. Hepatic infusion tube placement was performed according to the Seldinger method. The tip was placed at the gastroduodenal artery, and the drug was administered into the hepatic artery via the catheter side hole. A coiling procedure was performed to prevent drug influx into the gastroduodenal artery

Hepatic infusion tube placement was performed according to the Seldinger method in order to insert a catheter with a side hole via the right femoral artery; the tip was placed at the gastroduodenal artery, and the drug was administered into the hepatic artery via the catheter side hole. A coiling procedure was performed to prevent drug influx into the gastroduodenal artery (Fig. 1). Angiography was performed to allow drug influx into the hepatic artery. The chemotherapy regimen comprised once weekly 5-FU (500 mg/m^2^ for 5 min) and LV (200 mg/m^2^ for 2 h). Four 6-week courses were administered. The efficacy was evaluated using computed tomography (CT) after four courses; the efficacy was evaluated in accordance with the Response Evaluation Criteria in Solid Tumors, and adverse reactions were assessed according to assessed on the basis of RECIST (version 1.1) criteria [16] and National Cancer Institute Common Terminology Criteria for Adverse Events version 4.0 [17].

**Fig. 2:**
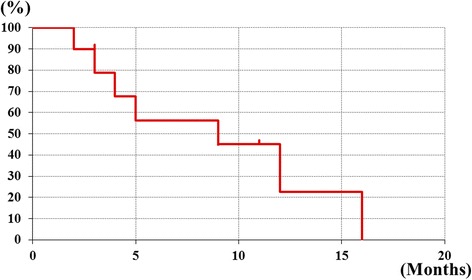
Overall survival curves of patients with unresectable hepatic metastases and impending hepatic failure treated with HAIC therapy. HAIC group had a 45 % rate of surviving 10 months, and median survival time(MST) is 9 months

### Chemicals

5-FU was dissolved in distilled water, and the pH was adjusted to 7.0 according to the manufacturer’s recommendation (Sigma Chemical Co. MO, USA).

### Resistant cell lines

Colon cancer cells were seeded onto a 6-cm dish at 1×10^5^ and incubated for 24 h. The cells were treated with 5-FU of 0.1 microgram/ml for 7 days. We established 5-FU-resistant cell lines.

### 5-FU treatment

5-FU-resistant colon cancer cells were seeded onto a 24-well plate at 1×10^4^ and incubated with 5-FU for 24 h.

### Cell viability

Cell death and apoptosis was detected by cytometry using Annexin-V-FLUOS staining Kit (Roche, Germany). After cells were washed in PBS, we detected red cells under a fluorescent microscope.

### Statistical analyses

Survival time was estimated by using Kaplan-Meier method. The characteristics of two treatment arms were evaluated by *t* test. Two-sided *p* < 0.05 were considered significant.

## Results

### Efficacy

Of the 10 subjects, 3 (30 %) showed partial response and 4 (40 %) showed no change according to computed tomography imaging findings obtained after hepatic artery infusion. The disease control rate was 70 %. A biochemical blood evaluation prior to hepatic artery infusion revealed abnormal alanine aminotransferase, aspartate aminotransferase, lactate dehydrogenase, and alkaline phosphatase levels in all of the subjects, indicative of hepatic dysfunction. After hepatic artery infusion, the abnormal hepatic function levels improved in all of the subjects (Table 2 and 3).

### Subjects’ performance statuses

Regarding the subjective symptoms, six patients experienced alleviation of leg edema. In particular, one subject who had difficulty walking was able to walk and perform housework. Four subjects experienced improvements in or disappearance of symptoms (Table 4).

### Survival time

The survival time ranged from 2 to 16 months (median, 9 months). An extrahepatic metastatic lesion was found in four patients after initiating hepatic artery infusion; this lesion did not affect the prognosis.

### Adverse reactions

Grade 1 anorexia and neutropenia were observed, but neither was serious. The symptoms of systemic malaise and anorexia that were experienced by the nine subjects during systemic chemotherapy were alleviated during the course of hepatic artery infusion (Table 5).

### Annexin V staining of the 5-FU-resistant colon cancer cell line exposed to high 5-FU concentrations

**Fig. 3:**
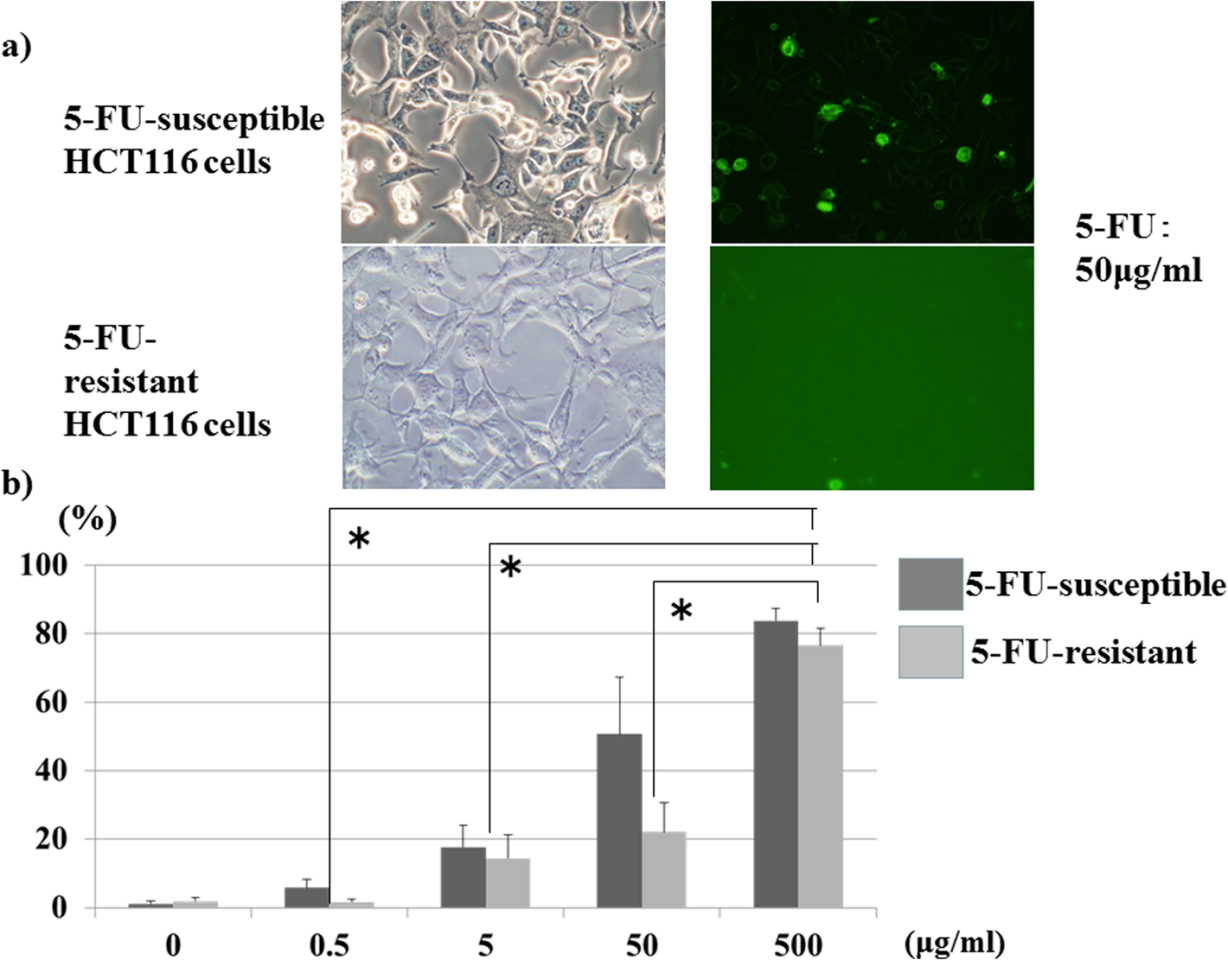
5-FU-resistant colon cancer cell line exposed to high 5-FU concentrations. **a** 5-FU-resistant HCT116 colon cancer cells (1 × 10^4^) were seeded onto 24 wells and were incubated with 5-FU for 24 h. Cell death and apoptosis was detected by cytometry using Annexin-V-FLUOS staining Kit. After washing cancer cells, the staining cell number were counted at ×200 magnification. *Left panel*: The cells were photographed at ×200 magnification. *Right panel* ANNEXIN-V-FLUOS staining cells. **b** The number of stained cells. Each *bar* represents the mean number of stained cells; *error bar* is SD. *p < 0.01

A 5-FU (0.01 μg/mL)-resistant colon cancer cell line was exposed to 0.5–500 μg/mL 5FU and stained according to the annexin V method in order to determine the number of apoptotic cells. The results are shown in Fig. 3. The number of stained cells increased significantly along with increased 5-FU concentrations in all of the 5FU-resistant colon cancer lines, possibly indicating apoptosis or cell death. However, the numbers of stained cells at any concentration were fewer than those observed in populations of 5-FU-susceptible colon cancer cells.

## Discussion

Hepatic metastasis is considered a prognosis-determining factor of colorectal cancer [2, 18]. Hepatic metastasis is observed in approximately 10 % of initial surgery cases, and 4 % of such cases are found to have multiple metastases throughout the liver [19, 20]. According to recent reports, the survival duration of liver metastatic colorectal cancer patients is prolonged when curative surgery is possible for not only the primary lesion but also the hepatic metastases [21–23]. However, colorectal cancers with multiple metastases throughout the liver must be treated via chemotherapy. Although systemic and arterial administrations are possible chemotherapeutic dosing routes, systemic administration has been recommended in the NCCN guideline and has therefore been selected in many clinical cases [5]. Nevertheless, no effective therapies are available for chemotherapy-resistant cases. Among such cases, patients for whom hepatic metastasis represented the greatest prognosis-determining risk factor were selected as subjects in the present study in order to examine the effects of hepatic artery infusion. The following are explanations of the pharmacological efficacy of hepatic artery infusion: (1) because higher drug concentrations can reach the tumor, compared with those achieved via intravenous infusion, artery infusion acts more effectively against tumor cells; (2) because smaller drug amounts enter non-tumor tissues throughout the body, the drug distribution in the organs other than the liver is reduced [24], possibly leading to fewer adverse reactions and higher maximum tolerable dosages. Hepatic arterial infusion is thought to be more effective than systemic administration for the treatment of liver diseases.

In the present study, disease control was temporarily achieved in seven of 10 cases of systemic chemotherapy-resistant unresectable colorectal cancers with hepatic metastasis and impending hepatic failure. Furthermore, subjective symptoms were improved, thus yielding a higher QOL and suggesting the effectiveness of this therapy. The incidence and grades of the adverse reactions were lower in comparison compared with systemic chemotherapy and the FOLFIRI and FOLFOX regimens.

The greatest cause of metastatic lesion growth inhibition in response to hepatic arterial infusion in the study patients under the described conditions was the increased drug concentrations in the target organ, in light of the experimental finding that higher concentrations of anticancer drugs could induce apoptosis and death in an anticancer drug-resistant cancer cell line.

## Conclusions

The efficacy of hepatic arterial infusion was observed in unresectable colorectal cancer patients with prognosis-determining hepatic metastases that were resistant to molecular-targeted agents and the FOLFOX and FOLFIRI regimens. This concept supported the finding that apoptosis and cell death could be induced in anticancer drug-resistant colorectal cancer cells in a drug concentration-dependent manner.

### Ethics

The procedures of our study received ethical approval with institutional committee responsible for human experimentation at the University of Fukui, and all those who participated in our study did so voluntarily, having given their informed consent.
